# Case Report: Complex atrial arrhythmia ablation with transbaffle puncture in a Fontan patient with intracardiac lateral tunnel anatomy

**DOI:** 10.3389/fcvm.2026.1792096

**Published:** 2026-04-08

**Authors:** Zhandos Maksut, Zhandos Esilbaev, Abay Bakytzhanuly, Abdurashid Mussayev, Yerlan Turubayev, Sardor Yuldashov, Ainabekova Bayan, Omirbek Nuralinov

**Affiliations:** 1 Interventional Arrhythmology Department, “University Medical Center” Corporate Fund, Astana, Kazakhstan; 2 Catheterization Laboratory Department, “University Medical Center” Corporate Fund, Astana, Kazakhstan; 3 Department of Internal Medicine, NJSC “Astana Medical University”, Astana, Kazakhstan

**Keywords:** atrial flutter, atrial tachycardia, catheter ablation, congenital heart disease, electrophysiological study, Fontan procedure, lateral tunnel, transbaffle puncture

## Abstract

Atrial arrhythmias are frequent and potentially life-threatening late sequelae in adults with Fontan physiology. We present the case of a 36-year-old woman with an intracardiac lateral tunnel Fontan and prior Amplatzer Septal Occluder closure of a residual fenestration, who developed recurrent, drug-refractory supraventricular tachycardia. High-density electroanatomical mapping revealed a macro-reentrant flutter circuit within the conduit, successfully interrupted with linear ablation. Preprocedural cardiac CT guided a transbaffle puncture, performed inferior and right to the occluder, enabling common atrial access. Subsequent mapping identified an additional macro-reentrant flutter and a focal atrial tachycardia, both ablated with restoration of stable sinus rhythm. No arrhythmias were inducible post-procedure. This case underscores the feasibility and safety of catheter ablation in complex Fontan anatomy when guided by meticulous anatomical assessment, tailored access, and advanced mapping technologies.

## Introduction

Since its introduction in 1971, the Fontan procedure has undergone significant evolution, with surgical modifications aimed at optimizing hemodynamics and minimizing late complications (1). The original atriopulmonary connection, which created a direct anastomosis between the right atrium and pulmonary artery, was associated with progressive atrial dilation and a high burden of atrial arrhythmias (2, 3). To address these limitations, the lateral tunnel modification was developed, incorporating a baffle within the right atrium to direct inferior vena cava (IVC) blood flow to the pulmonary arteries, often with a fenestration to decompress elevated venous pressures (2, 4). More recently, the extracardiac conduit technique has become the preferred strategy, using an external conduit to bypass atrial tissue and reduce arrhythmogenic substrate (4, 5).

Despite these advances, atrial arrhythmias remain a frequent late complication, particularly in patients with intracardiac pathways such as the lateral tunnel (6, 7). Progressive atrial remodeling, surgical suture lines, and scarring contribute to complex arrhythmogenic substrates (6, 8).

Antiarrhythmic therapy often provides limited benefit due to altered atrial anatomy and abnormal drug kinetics (9). Catheter ablation, guided by three-dimensional electroanatomical mapping, has emerged as a cornerstone in managing both macro-reentrant and focal atrial tachyarrhythmias, with reported acute success rates of 65%–100%, though recurrences remain common, approaching 50%–70% over two years (7, 10). Procedural challenges are amplified by complex anatomy, conduits, fenestrations, and implanted devices, which can hinder catheter access and mapping (11, 12).

Recent reports highlight the feasibility and clinical benefits of ablation in this cohort, particularly when combined with advanced imaging, high-density mapping, and tailored strategies addressing multiple circuits (7, 10, 13). This case demonstrates such an approach, achieving effective ablation of macro-reentrant and focal arrhythmias in a single procedure, with added complexity due to the need for transbaffle puncture and the presence of multiple arrhythmogenic mechanisms (6, 7, 10).

## Case presentation

We report the case of a 36-year-old woman (weight: 80 kg, height: 168 cm) with complex congenital heart disease (CHD), including single ventricle, transposition of the great arteries, pulmonary artery stenosis. A lateral tunnel (intracardiac conduit) Fontan procedure was performed at the age of 20, and two years later the residual fenestration was closed percutaneously using an Amplatzer Septal Occluder.

The patient presented with frequent, symptomatic paroxysms of tachycardia with a narrow QRS complex, with heart rate 180 bpm, accompanied by mild dyspnea and dizziness, despite treatment with low-dose bisoprolol (2.5 mg daily). Amiodarone was not prescribed due to the patient's predisposition to arterial hypotension, and class I antiarrhythmic agents were contraindicated given the presence of significant structural heart disease.

Baseline laboratory investigations were within normal limits. Transthoracic echocardiography demonstrated a patent intracardiac Fontan tunnel with preserved respiratory phasic flow and no evidence of residual shunting. The inferior vena cava measured 1.61 cm and exhibited >50% inspiratory collapse, suggesting the absence of elevated Fontan pathway pressures. Mild systemic atrioventricular valve regurgitation was present (grade 1–1.5+). The systemic single ventricle was dilated, with an end-diastolic diameter of 6.27–6.40 cm and an end-systolic diameter of 5.64 cm. Longitudinal systolic function was reduced, as reflected by TAPSE 1.27 cm, MAPSE 1.19 cm, dp/dt 638 mmHg/s, and lateral S’ velocity 7.1 cm/s, consistent with moderately impaired systemic ventricular systolic function.

**Figure 1:**
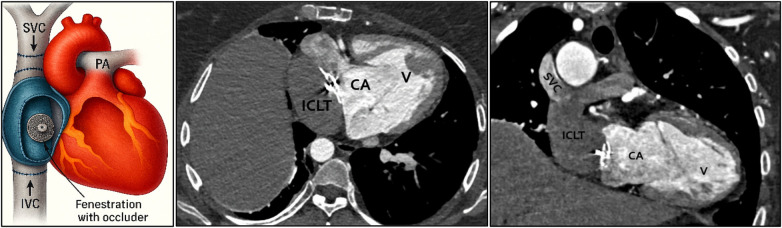
Representation of an intra-atrial lateral tunnel (ICLT) Fontan circulation. Systemic venous blood from the superior vena cava (SVC) and inferior vena cava (IVC) is directed to the pulmonary arteries (PA) through a surgically constructed baffle within the right atrium. A fenestration between the tunnel and the common atrium (CA) is illustrated, serving as a pressure-relief mechanism with potential for right-to-left shunting. In this patient, the fenestration was subsequently closed percutaneously using an occluder following the Fontan procedure.

To further delineate the complex cardiac anatomy prior to intervention, contrast-enhanced cardiac computed tomography (CT) was performed, allowing detailed visualization of the Fontan pathway, atrial morphology, and the position of the Amplatzer septal occluder (Figure 1).

Antiarrhythmic medications were discontinued five days prior to the procedure. Informed consent was obtained. The procedure was conducted under local anesthesia using 20 mL of 2% lidocaine.

**Figure 2:**
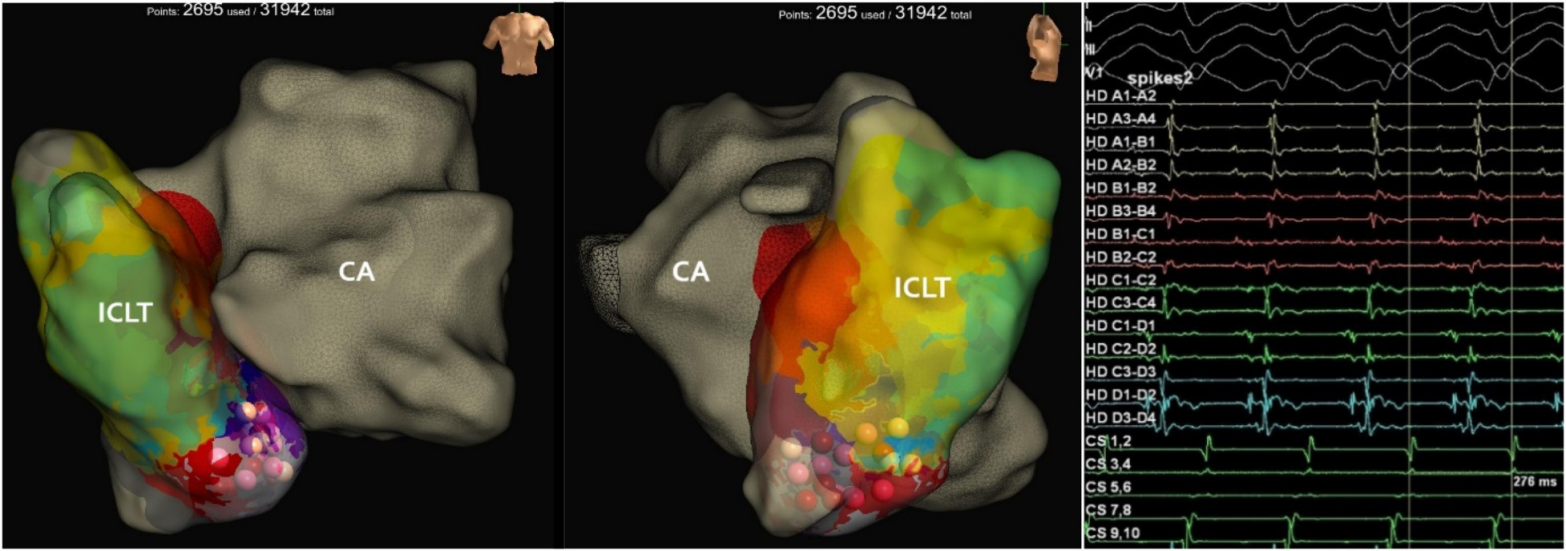
Three-dimensional activation map of the intracardiac tunnel during induced atrial flutter (cycle length 276 ms. The macro-reentrant circuit involves the lower portion of the conduit near the inferior vena cava, with early-meets-late activation highlighted. Ablation lesions (pink/red spheres) represent the linear lesion set delivered along this area. Intracardiac electrograms during the atrial flutter showing a cycle length of 276 ms prior to ablation. Fractionated electrograms with spiky, low-amplitude potentials are visible on the multipolar mapping catheter, suggesting the presence of areas of conduction delay or scar.

Vascular access was obtained via double puncture of the right femoral vein—one for placement of a 10-pole diagnostic catheter into the intracardiac tunnel to obtain the referent signal, and the other for introduction of a high-density catheter (HD Grid, Abbott, USA). Additionally, the left femoral artery was punctured for retrograde access, and a 4-pole diagnostic catheter was advanced via the aorta into the single ventricular chamber. After establishing vascular access, intravenous heparin (11,000 IU) was administered to achieve therapeutic anticoagulation; the activated clotting time (ACT) during procedure was >300 ms. During intracardiac electrophysiological study (EPS) sustained atrial flutter was induced at a cycle length of 276 ms (Figure 2). Three-dimensional activation map of the intracardiac tunnel created during induced atrial flutter by using the Ensite X™ system with the high-density catheter (HD Grid, Abbott, USA), and sequentially localized the reentrant circuit within the intracardiac tunnel. An early-meets-late zone was identified in the lower portion of the conduit (Figure 2). Linear radiofrequency catheter ablation (RFCA) was performed from the lower conduit to the IVC using an irrigated contact force-sensing ablation catheter (TactiCath™, Abbott, USA).

Following ablation targeting the initial atrial flutter substrate, sinus rhythm was not restored. Instead, the tachycardia cycle length gradually prolonged to 290 ms without termination.

Repeat activation mapping of the lateral tunnel was performed. However, mapping demonstrated activation accounting for only part of the tachycardia cycle length, without a complete early-meets-late pattern within the accessible Fontan pathway. No discrete critical isthmus or mid-diastolic fractionated electrograms were identified within the lateral tunnel that could explain the entire tachycardia cycle.

These findings suggested incomplete modification of the initial macroreentrant circuit or involvement of atrial myocardium outside the lateral tunnel. High-density mapping raised suspicion of macroreentrant activation involving the common atrium.

**Figure 3:**
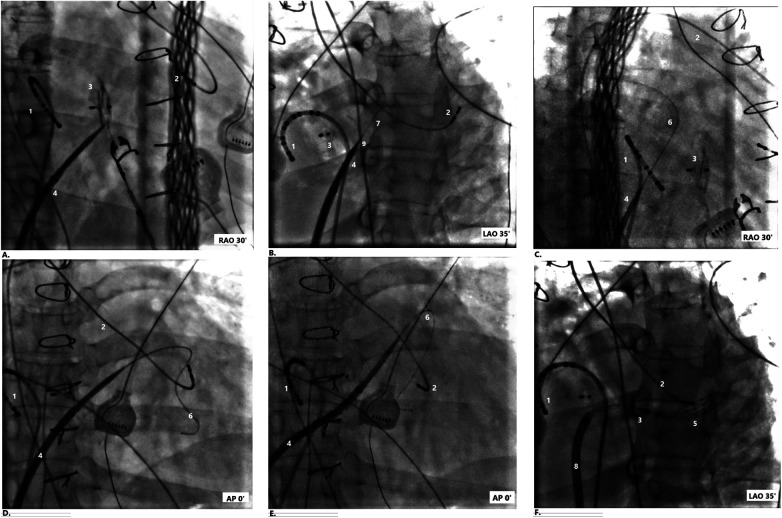
Fluoroscopic guidance during transbaffle puncture and common atrial mapping in a patient with lateral tunnel Fontan anatomy. Sequential projections illustrate key procedural steps. **(A)** Transbaffle puncture of the intracardiac conduit. Right anterior oblique (RAO) 30° projection. **(B)** Contrast injection through the transseptal needle to confirm common atrial entry. Left anterior oblique (LAO) 35° projection. **(C)** Advancement of the guidewire into the common atrium post-puncture. RAO 30° projection. **(D)** Advancement of the guidewire into the common atrium post-puncture. Anteroposterior (AP) 0° projection. **(E)** Advancement of the 8F introducer sheath across the transbaffle puncture site. AP 0° projection. **(F)** Common atrial activation mapping after exchanging the introducer sheath (Swartz, St. Jude Medical, USA) for a steerable sheath (Agilis™, Abbott). LAO 35° projection. Catheter and Device Labels: (1) 10-pole diagnostic catheter positioned in the intracardiac conduit. (2) 4-pole diagnostic catheter within the single ventricular chamber. (3) Occluder in the mid-conduit region. (4) Introducer sheath (Swartz, St. Jude Medical, USA) positioned for transbaffle puncture. (5) High-density catheter (HD Grid, Abbott, USA). (6) Guidewire advanced into the common atrium. (7) Contrast injected to confirm atrial entry. (8) Steerable sheath (Agilis™, Abbott) positioned for stable access to the common atrium.

**Figure 4:**
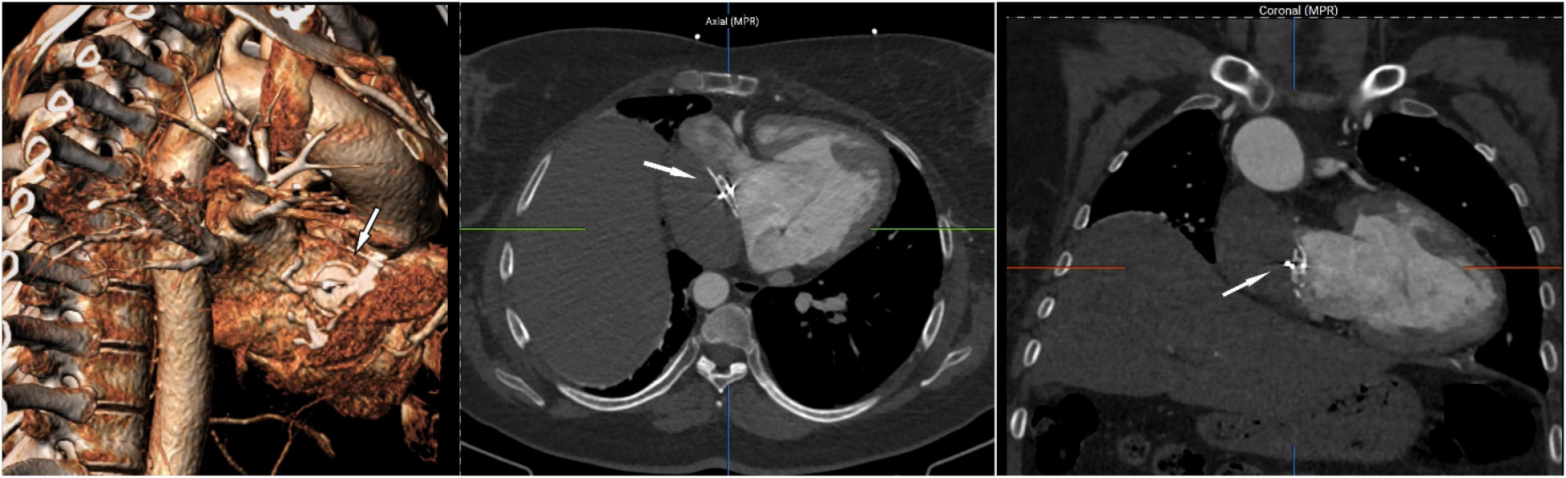
3D computed tomography assessment for optimal transbaffle puncture site selection relative to the occluder (arrows indicating the occluder).

Given the absence of a sustained reentrant circuit within the intracardiac tunnel on the prolonged flutter cycle, decided to perform transbaffle puncture (Figure 3) to access the common atrium (CA) for further mapping. Prior to performing the transbaffle puncture, the patient's preprocedural cardiac CT was reviewed in the operating room to delineate the occluder's anatomy and determine the optimal puncture site (Figure 4). A introducer sheath (Swartz, St. Jude Medical, USA) was advanced into the tunnel via the IVC, followed by insertion of a transseptal needle. The occluder served as a key fluoroscopic landmark. Based on CT reconstruction and fluoroscopic orientation, the puncture site was selected inferior and to the right of the occluder device, targeting a region presumed to provide direct access to the common atrium while avoiding the device margins and minimizing the risk of extracardiac perforation. Contrast injection confirmed entry into the common atrial cavity. A long guidewire was positioned within the common atrium following successful puncture. Transbaffle access was obtained by direct advancement of an 8F long introducer sheath (Swartz, St. Jude Medical, USA) over the guidewire. This was subsequently exchanged for an 8.5F steerable sheath (Agilis™, Abbott) to optimize catheter stability during mapping and ablation. The maximum diameter across the transbaffle tract was 8.5F.

**Figure 5:**
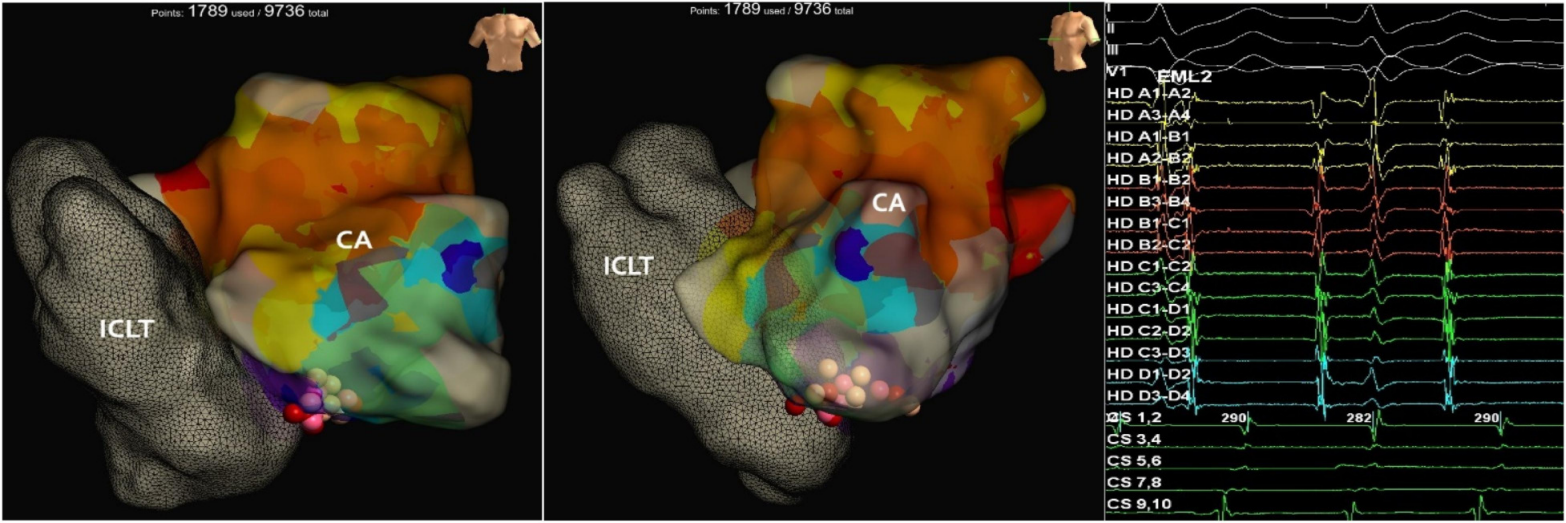
Three-dimensional activation map of atrial flutter with a prolonged cycle length of 290 ms following initial linear ablation. The reentrant circuit involves the region between the mitral annulus and the inferior aspect of the common atrium. Ablation lesions (colored spheres) indicate the linear lesion set that resulted in flutter termination.

**Figure 6:**
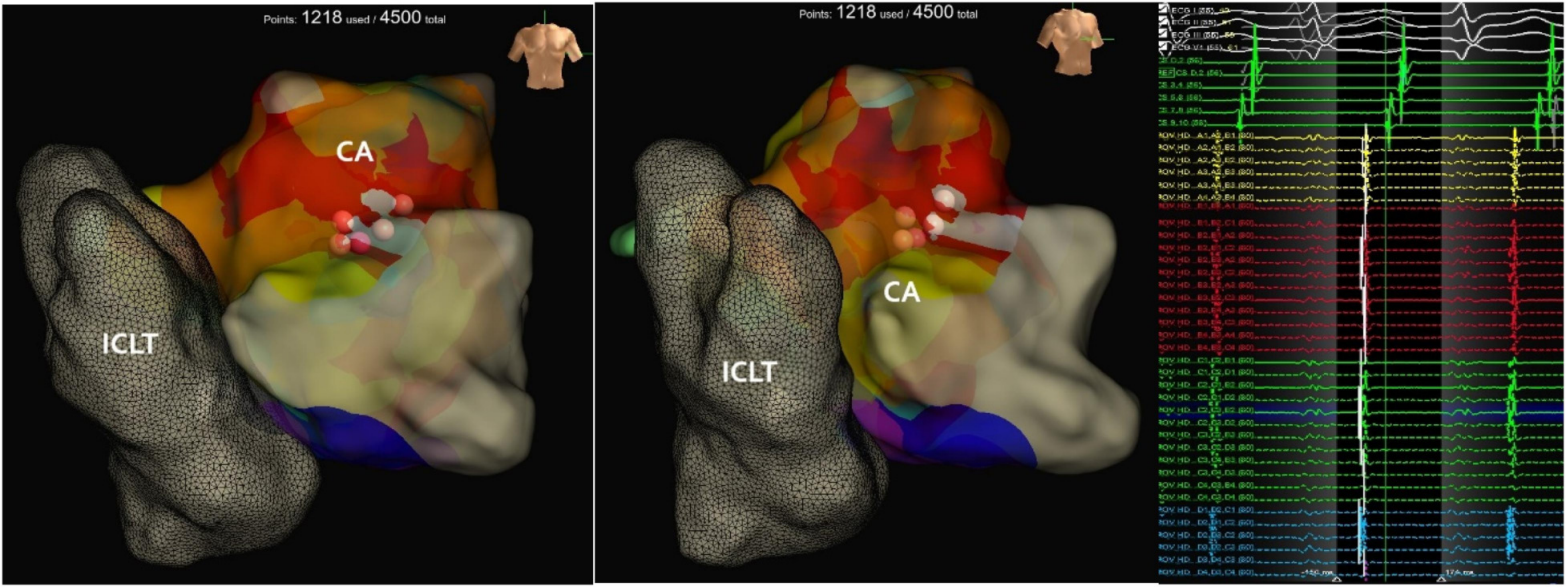
Intracardiac electrograms during induced focal atrial tachycardia at approximately 130 bpm, demonstrating early activation in the common atrium. Focal ablation at this site successfully restored sinus rhythm.

Activation mapping of the common atrium identified a macro-reentrant circuit involving the region between the mitral annulus and the lower conduit (Figure 5). Linear RF ablation was performed from the mitral annulus to the inferior part of the conduit, successfully terminating the atrial flutter and restoring sinus rhythm at a rate of 65 bpm. Subsequent rapid atrial pacing induced a focal atrial tachycardia (AT) at a rate of 130. High-density mapping localized the earliest activation to the lower third of the conduit (Figure 6). Focal RF ablation at this site resulted in immediate termination of the tachycardia and restoration of stable sinus rhythm.

Following a 20-minute observation period with programmed stimulation and rapid atrial pacing, no further arrhythmias were inducible. The procedure was completed without complications, and the patient was discharged in stable sinus rhythm at 74 bpm.

Following catheter and sheath removal, the puncture site was assessed by postoperative transthoracic echocardiography. A small residual fenestration (approximately 2–2.5 mm) was identified on color Doppler imaging, with predominantly right-to-left shunting.

In the absence of clinically significant hypoxemia or hemodynamic compromise, and considering the potential pressure-relief role of small fenestrations in Fontan circulation, the defect was not closed.

Postprocedural anticoagulation strategy was as follows: oral anticoagulation was resumed 6 h after sheath removal; the patient was continued on rivaroxaban (20 mg once daily); long-term anticoagulation was maintained given Fontan physiology and atrial arrhythmia history.

## Discussion

This case highlights the inherent complexity of atrial arrhythmia mechanisms in adults with Fontan circulation and underscores the necessity of a systematic, substrate-guided electrophysiological approach. The integration of advanced three-dimensional electroanatomical mapping system EnSite™ X (Abbott, USA) with high-density multipolar mapping (HD Grid, Abbott, USA) enabled precise delineation of both macro-reentrant and focal arrhythmogenic circuits within surgically modified cardiac structures. Notably, the arrhythmogenic substrates were localized within the intracardiac conduit and common atrium, emphasizing the importance of individualized mapping strategies tailored to the patient's unique anatomy.

A stepwise procedural strategy, involving sequential mapping of the Fontan tunnel and common atrium, facilitated accurate identification of arrhythmia circuits and effective ablation targeting. Preprocedural cardiac computed tomography (CT) played a pivotal role in anatomical assessment, particularly in delineating the spatial orientation of prior surgical modifications and transcatheter implants, such as the occluder. Safe transbaffle access, which can be challenging in the presence of intracardiac implants, was achieved through meticulous imaging review and operator expertise, underscoring the critical role of preprocedural planning in adult congenital heart disease (ACHD) interventions.

The coexistence of multiple arrhythmia mechanisms—including macro-reentrant flutter and focal atrial tachycardia—within a single patient reinforces the need for comprehensive, multipoint mapping and a flexible ablation strategy. The successful elimination of both arrhythmias in a single session demonstrates the feasibility and efficacy of catheter ablation in this high-risk population. Beyond rhythm control, arrhythmia resolution contributed to symptomatic relief and may favorably impact Fontan hemodynamics and long-term functional status.

This case aligns with the growing body of evidence supporting catheter ablation as a cornerstone of arrhythmia management in adults with Fontan physiology. However, the progressive nature of Fontan-associated circulatory decline, coupled with ongoing atrial remodeling, necessitates vigilant long-term follow-up to monitor for arrhythmia recurrence and Fontan failure. Prospective studies are required to refine patient selection, define optimal procedural timing, and develop standardized ablation strategies in this anatomically complex cohort.

## Limitations

As a single-case report, the generalizability of these findings is inherently limited. The anatomical variability among Fontan patients and the individualized procedural strategies required pose challenges to establishing uniform ablation protocols. Moreover, long-term arrhythmia-free survival remains to be determined, emphasizing the need for extended follow-up. Procedural success in this case was also facilitated by high operator expertise and advanced imaging capabilities, which may not be universally available in all electrophysiology centers.

## Scientific implications

This case report contributes valuable insights into the procedural intricacies of catheter ablation in patients with lateral tunnel Fontan circulation, particularly in scenarios involving prior transcatheter interventions. It demonstrates the feasibility of transbaffle puncture in the presence of intracardiac implants and highlights the utility of high-density mapping technologies in complex ACHD substrates. These findings may inform procedural planning and serve as a reference for future studies aiming to optimize ablation strategies in this challenging patient population.

## Conclusion

Catheter ablation represents a safe and effective strategy for rhythm control in adult Fontan patients with complex atrial arrhythmias. The use of advanced mapping technologies combined with individualized ablation approaches enables precise targeting of both macro-reentrant and focal circuits. Thorough anatomical assessment is critical to procedural planning and success in this challenging population.

## Data Availability

The raw data supporting the conclusions of this article will be made available by the authors, without undue reservation.
